# Common Expression Quantitative Trait Loci Shared by Histone Genes

**DOI:** 10.1155/2017/6202567

**Published:** 2017-08-27

**Authors:** Hanseol Kim, Yujin Suh, Chaeyoung Lee

**Affiliations:** Department of Bioinformatics and Life Science, Soongsil University, Seoul, Republic of Korea

## Abstract

A genome-wide association study (GWAS) was conducted to examine expression quantitative trait loci (eQTLs) for histone genes. We examined common eQTLs for multiple histone genes in 373 European lymphoblastoid cell lines (LCLs). A linear regression model was employed to identify single-nucleotide polymorphisms (SNPs) associated with expression of the histone genes, and the number of eQTLs was determined by linkage disequilibrium analysis. Additional associations of the identified eQTLs with other genes were also examined. We identified 31 eQTLs for 29 histone genes through genome-wide analysis using 29 histone genes (*P* < 2.97 × 10^−10^). Among them, 12 eQTLs were associated with the expression of multiple histone genes. Transcriptome-wide association analysis using the identified eQTLs showed their associations with additional 80 genes (*P* < 4.75 × 10^−6^). In particular, expression of RPPH1, SCARNA2, and SCARNA7 genes was associated with 26, 25, and 23 eQTLs, respectively. This study suggests that histone genes shared 12 common eQTLs that might regulate cell cycle-dependent transcription of histone and other genes. Further investigations are needed to elucidate the transcriptional mechanisms of these genes.

## 1. Introduction

Histone mRNA transcripts and proteins are important for packing DNA into chromatin and are thus tightly regulated in most human cells [1]. In humans, the genes encoding histones are gathered on chromosomes 1 and 6. It has been suspected that the clustered structure of genes can provide a manageable unit for coordinating transcription [1]. Recently, genome-wide chromatin interaction analysis with paired-end-tag sequencing (ChIA-PET) has shown that some histone genes can share promoters [2].

While many efforts have been made to understand the mechanisms for the transcription of histone genes, they have not yet been well defined. Nuclear protein of the ataxia-telangiectasia-mutated locus (NPAT), which promotes the transcription of histone genes, is located near the Cajal body [1]. Clusters of histone genes are also located near the Cajal body [3]. The positions of histone gene clusters near the Cajal body have been observed between the restriction point (R-point) and the G1/S transition (S-point) during the cell cycle [4]. The objective of this study was to select simultaneously expressed histone genes, identify their expression quantitative trait loci (eQTLs), and examine the functions of those eQTLs.

## 2. Material and Methods

### 2.1. Subjects and Data

The subjects of this study were 373 Europeans including 95 Finnish in Finland, 94 British in England and Scotland, 93 Tuscans from Italy, and 91 Utahn residents with Northern and Western European ancestry from the CEPH collection. Their genotypic data were derived from the phase 1 dataset produced by the 1000 Genomes Project [5] (http://www.internationalgenome.org/). This study utilized genotypic data at 5,796,145 SNPs after filtering out the SNPs with minor allele frequency < 0.05, with missing rate > 0.05, or in Hardy-Weinberg disequilibrium with *P* < 0.001.

Transcriptional data on 10,518 human genes were obtained in lymphoblastoid cells of the subjects by the Geuvadis RNA sequencing project (http://www.geuvadis.org/web/geuvadis/rnaseq-project). The unit used for the mRNA expression level was reads per kilobase per million mapped reads (RPKM). Outliers were removed based on sample similarity, which was estimated by the Spearman rank correlation between RPKMs and the exon counts of the samples [6]. Sample swaps or contaminated samples were excluded based on allele-specific expression analysis [6]. For details on the quality control process, see t Hoen et al. [7].

### 2.2. Statistical Methods

We selected histone genes that were expressed simultaneously. Pairwise gene expression relationships were estimated using Pearson's correlation coefficient (*r*). The significance of the correlation was determined by *P* < 0.05.

We investigated genome-wide associations of the expression of the selected histone genes. A regression model was employed to identify SNPs associated with expressions of histone genes using PLINK [8]. The Bonferroni correction was applied as a multiple testing, and the significance was determined by *P* < 2.97 × 10^−10^.

Linkage disequilibrium (LD) between the identified SNPs was estimated using the HaploView program [9]. The LD block was determined according to the 95% confidence interval of the *D*′ value for pairwise LD between the nucleotide variants with minor allele frequency > 0.05 [10].

The identified eQTLs were further analyzed for their associations with the expression of nonhistone genes throughout the genome. The Bonferroni multiple testing based on *t*-statistic was also applied with a significance threshold value of *P* = 4.75 × 10^−6^.

The functions of identified SNPs were examined using the Ensembl Variant Effect Predictor program [11] and RegulomeDB [12] (e.g., the motif of DNA footprinting assay, chromatin structure by DNA-seq, and protein binding by ChIP-seq).

## 3. Results

**Figure 1:**
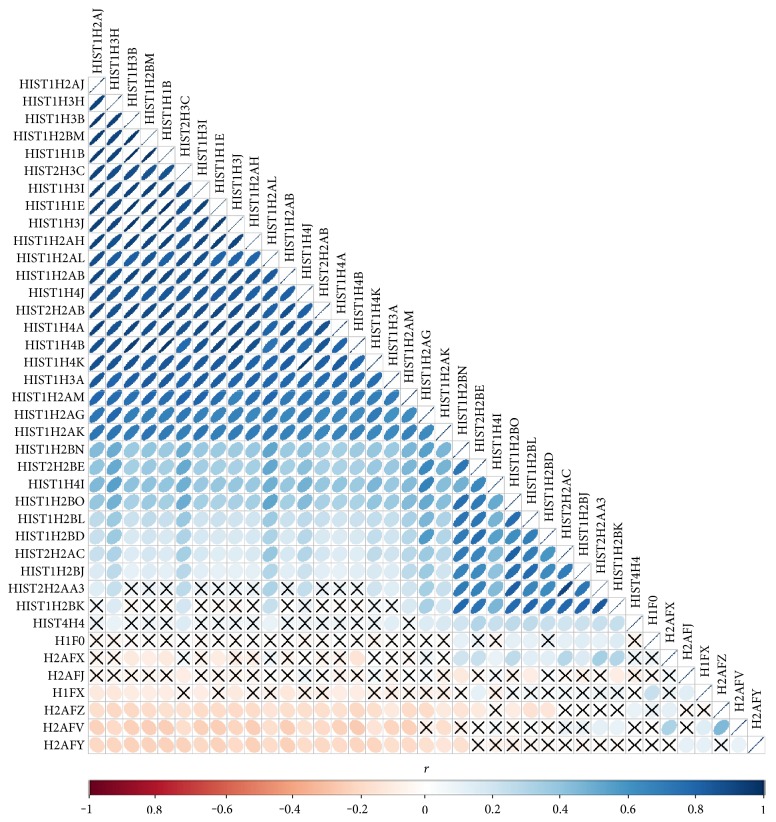
Pearson's correlation (*r*) between expressions of histone genes using the ellipse visualization method. The correlation estimate without significance (*P* > 0.05) is marked with “X.”

**Figure 2:**
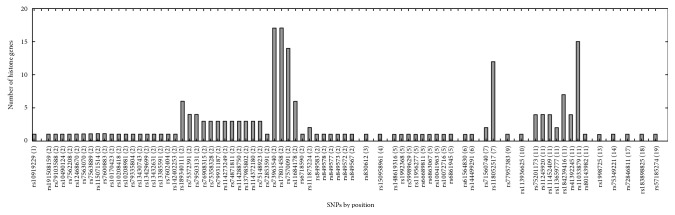
Number of histone genes with expression associated with SNPs. The figure in parentheses following the SNPs indicates the chromosomal number.

**Figure 3:**
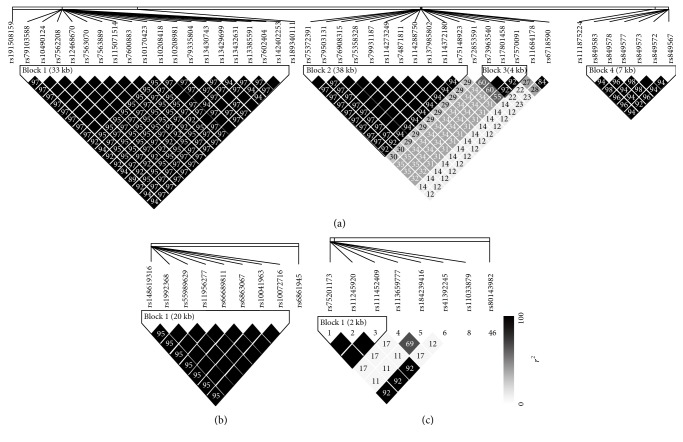
Linkage disequilibrium (LD) blocks of SNPs associated with the expression of histone genes. (a) 4 LD blocks in chromosome 2 (48891009-48924921, 141415941-141454893, 141710041-141714131, and 205737518-205744540), (b) 1 LD block in chromosome 5 (14080602-14101222), and (c) 1 LD block in chromosome 11 (1062030-1064929). No LD blocks were observed in chromosomes 6 and 7.

We observed numerous correlations amid the expression of the histone genes investigated in the current study (Figure 1). In particular, the expression of 29 genes showed correlations significantly (*P* < 0.05). Genome-wide association analysis showed that 74 SNPs were associated with the expression of the 29 histone genes (*P* < 2.97 × 10^−10^; Table 1). Among them, 26 SNPs were simultaneously associated with the expression of multiple histone genes, and 5 out of 26 SNPs were associated with the expression of more than 10 histone genes (Figure 2). Thirty-one LD blocks were constructed covering the identified SNPs (Figure 3). The eQTLs corresponded to functional sites provided by various functional search sites. The rs79335804 had the most probable function with a RegulomeDB score of 2b (Table S1 available online at https://doi.org/10.1155/2017/6202567).

Transcriptome-wide association analysis revealed 80 additional genes associated with the 31 identified eQTLs (*P* < 4.75 × 10^−6^; Table 2). The genes encoding ribonuclease P RNA component H1 (RPPH1) and some small Cajal body-specific RNAs (scaRNAs), in particular, were associated with more than half of the eQTLs (>15 eQTLs; Table 2).

## 4. Discussion

We analyzed the eQTLs for simultaneously expressed histone genes. We found significant correlations amid the expression of 29 histone genes, which were all clustered in chromosome 1 or 6. This clustered structure of the genes may serve to control simultaneous transcription, and this is supported by the observation that the expression of other histone genes not located on chromosome 1 or 6, including H1FX and H2A family members, was not correlated with those of the 29 selected genes. Furthermore, correlation estimates showed two subgroups nested within the large group (one with 21 genes and the other with 10 genes; with strong correlation coefficients of *r* > 0.7), which likely provide a manageable unit for coordinating transcription.

The genome-wide eQTL analysis revealed that 12 loci were associated with the expression of multiple histone genes. The eQTLs were located on chromosomes 2, 7, and 11. Since 29 histone genes were all located on chromosome 1 or 6, we suspect that the identified eQTLs were transacting. This suggests that many histone genes are simultaneously transcribed by remote regulators.

Functional analysis of the identified eQTLs suggests that they are very important for transcription. For example, rs79335804, an SNP within an eQTL on chromosome 2, was the binding motif for Kruppel-like factor 4 (Klf4) protein in various cells including LCLs. Klf4 was associated with chromosomal aberrations and can prevent cell proliferation by acting as a transcription factor [13]. The aberrant chromatin formation could be caused by overproduction of a histone dimer set (H2A-H2B or H3-H4) [14]. Thus, we suspect that there is an association between the chromosomal aberrations by Klf4 and histone gene mRNA expression. Rs849578 within another eQTL on chromosome 2 was associated with autism in the Chinese Han population [15]. It is located in an intron of neuropilin 2 (NRP2) which may be an effector of apoptosis, proliferation, and neuronal development [16]. Histones are known to be related to developmental regulation [17], but additional study is required to elucidate underlying mechanism of the relationship between histones and NRP2.

Transcriptome-wide association analysis revealed that many nonhistone genes were also associated with the identified eQTLs. In particular, some genes were associated with 23 or more eQTLs. One was RPPH1, an RNA component of RNase P, which may assist in the cell cycle-dependent transcription of ribosomal RNAs (rRNAs) by associating with chromatin [18]. The expression of rRNAs increased from G1 to S and peaked at G2 [18]. The transcription of histone genes rapidly increased before the S phase of the cell cycle and decreased shortly thereafter [1]. Thus, many eQLTs identified in this study might be involved in the cell cycle-dependent expression of both RPPH1 and histone genes. Such a regulation of the eQTLs would be one of the key factors to solve their underlying mechanisms. The others identified with many eQTLs were the genes encoding scaRNAs (SCARNA2 and SCARNA7) located in the Cajal body, similar to the pre-mRNAs of histones, which move to the Cajal body for mRNA processing [3, 19].

Interestingly, many genes controlled by the same eQTLs as those for histones do not have polyadenylated structures [20]. In particular, the genes associated with more than 10 eQTLs were all nonpolyadenylated. They were snoRNAs, scaRNAs, and RPPH1. Considering that histones are also nonpolyadenylated, this may help us to understand the transcriptional regulation of histone genes by these eQTLs.

The expressions of histone genes play an important role in controlling chromatin accessibility [21]. Improper expression of histone genes has been associated with tumorigenesis [22–24]. Expression of NPAT, a transcriptional activator for histone genes, is also associated with human tumorigenesis [25]. The influence of histone genes on tumor developments might be supported by the eQTLs identified in the current study, because some of the eQTLs were located within anticipated tumor suppressor genes such as low-density lipoprotein receptor-related protein (LRP1B) and utrophin (UTRN) [26, 27].

In conclusion, we identified 31 eQTLs for histone genes. The eQTLs were also associated with nonhistone genes that exhibited both a cell cycle-dependent expression and a nonpolyadenylated RNA structure. Further investigations are required to understand the mechanisms regulating the transcription of the histone and nonhistone genes identified in this study and to appreciate their influence on cancer and other diseases. Moreover, identification of eQTLs using disease-specific cell types would provide resolute mechanisms by diseases.

## Supplementary Material

Table S1. Functional capability of eQTLs identified for histones using RegulomeDB.

## Figures and Tables

**Table 1:** Genome-wide associations of SNPs with expression of histone genes.

SNP^a^	Gene	Effect^b^	Location^c^	A1^d^	A2^d^	MAF	Genetic associations	Beta	*P*
**rs10919229**	SELE (2Kb upstream)		1:169735986	T	A	0.05	HIST1H4B	1.035	2.51*E*−10

**rs191508159**	Intergenic		2:35957035	T	C	0.06	HIST1H4A	0.466	3.41*E*−11

rs79103588	Intergenic		2:48891009	A	T	0.05	HIST1H4B	1.045	1.02*E−*10

rs10490124	Intergenic		2:48897335	G	A	0.06	HIST1H4B	1.023	1.65*E−*10

rs7562208	Intergenic		2:48898231	C	T	0.06	HIST1H4B	1.023	1.65*E−*10

rs12468670	Intergenic		2:48902054	T	G	0.06	HIST1H4B	1.023	1.65*E−*10

rs7563070	Intergenic		2:48902202	T	C	0.06	HIST1H4B	1.023	1.65*E−*10

rs7563889	Intergenic	CTCF	2:48902751	T	G	0.06	HIST1H4B	1.023	1.65*E−*10

rs115071514	Intergenic		2:48902928	T	C	0.06	HIST1H4B	1.023	1.65*E−*10

rs7600883	Intergenic		2:48905331	G	A	0.06	HIST1H4B	1.023	1.65*E−*10

rs10170423	Intergenic		2:48906784	G	A	0.06	HIST1H4B	1.023	1.65*E−*10

rs10208418	Intergenic		2:48912244	A	T	0.05	HIST1H4B	1.079	3.82*E−*11

rs10208981	Intergenic		2:48912890	G	T	0.05	HIST1H4B	1.049	8.60*E−*11

rs79335804	Intergenic		2:48915424	T	C	0.05	HIST1H4B	1.079	3.82*E−*11

rs13430743	Intergenic		2:48916396	T	G	0.05	HIST1H4B	1.079	3.82*E−*11

rs13429699	Intergenic		2:48921530	G	C	0.05	HIST1H4B	1.049	8.60*E−*11

**rs13432631**	Intergenic		2:48921948	A	C	0.05	HIST1H4B	1.108	1.78*E−*11

rs13385591	Intergenic		2:48923227	T	C	0.05	HIST1H4B	1.079	3.82*E−*11

rs7602404	Intergenic		2:48923414	A	G	0.05	HIST1H4B	1.049	8.60*E−*11

rs142402253	Intergenic		2:48924921	A	G	0.05	HIST1H4B	1.049	8.60*E−*11

**rs189340111**	Intergenic		2:68450091	G	A	0.06	HIST1H4B	0.995	2.99*E−*12
							HIST1H1B	2.319	2.91*E−*11
							HIST1H1E	2.044	6.90*E−*11
							HIST1H3J	1.044	8.17*E−*11
							HIST1H2AH	0.745	2.34*E−*10
							HIST1H2BM	1.027	2.58*E−*10

**rs75372391**	LRP1B (intron 2)		2:141415941	G	A	0.05	HIST1H2BM	1.268	6.19*E−*12
							HIST1H4B	1.102	1.39*E−*11
							HIST1H1B	2.645	2.92*E−*11
							HIST1H3B	2.016	2.00*E−*10

rs79503131	LRP1B (intron 2)		2:141417401	T	C	0.05	HIST1H2BM	1.268	6.19*E−*12
							HIST1H4B	1.102	1.39*E−*11
							HIST1H1B	2.645	2.92*E−*11
							HIST1H3B	2.016	2.00*E−*10

rs76908315	LRP1B (intron 2)		2:141428575	G	A	0.05	HIST1H2BM	1.246	9.09*E−*12
							HIST1H4B	1.080	2.15*E−*11
							HIST1H1B	2.590	4.75*E−*11

rs75358328	LRP1B (intron 2)		2:141435763	C	T	0.05	HIST1H2BM	1.246	9.09*E−*12
							HIST1H4B	1.080	2.15*E−*11
							HIST1H1B	2.590	4.75*E−*11

rs79931187	LRP1B (intron 2)		2:141440679	C	T	0.05	HIST1H2BM	1.246	9.09*E−*12
							HIST1H4B	1.080	2.15*E−*11
							HIST1H1B	2.590	4.75*E−*11

rs114273249	LRP1B (intron 2)		2:141441139	T	C	0.05	HIST1H2BM	1.246	9.09*E−*12
							HIST1H4B	1.080	2.15*E−*11
							HIST1H1B	2.590	4.75*E−*11

rs74871811	LRP1B (intron 2)		2:141441981	T	A	0.05	HIST1H2BM	1.246	9.09*E−*12
							HIST1H4B	1.080	2.15*E−*11
							HIST1H1B	2.590	4.75*E−*11

rs114288750	LRP1B (intron 2)		2:141442588	A	C	0.05	HIST1H2BM	1.246	9.09*E−*12
							HIST1H4B	1.080	2.15*E−*11
							HIST1H1B	2.590	4.75*E−*11

rs137985802	LRP1B (intron 2)		2:141442673	T	C	0.05	HIST1H2BM	1.246	9.09*E−*12
							HIST1H4B	1.080	2.15*E−*11
							HIST1H1B	2.590	4.75*E−*11

rs114372180	LRP1B (intron 2)		2:141442782	A	G	0.05	HIST1H2BM	1.246	9.09*E−*12
							HIST1H4B	1.080	2.15*E−*11
							HIST1H1B	2.590	4.75*E−*11

rs75148923	LRP1B (intron 2)		2:141454893	T	C	0.05	HIST1H2BM	1.236	1.33*E−*11
							HIST1H4B	1.080	2.15*E−*11
							HIST1H1B	2.588	4.96*E−*11

**rs72853591**	LRP1B (intron 2)	OC	2:141557777	C	T	0.06	HIST1H4B	1.015	9.68*E−*11

rs73963540	LRP1B (intron 2)		2:141710041	T	C	0.05	HIST1H1B	2.975	1.62*E−*14
							HIST1H2BM	1.369	3.12*E−*14
							HIST1H3B	2.277	1.75*E−*13
							HIST1H4B	1.154	4.90*E−*13
							HIST1H2AB	0.310	8.94*E−*13
							HIST1H3J	1.273	1.28*E−*12
							HIST1H1E	2.439	3.33*E−*12
							HIST1H2AJ	1.711	4.01*E−*12
							HIST1H2AL	0.552	9.35*E−*12
							HIST2H2AB	0.263	1.43*E−*11
							HIST2H3C	0.609	4.10*E−*11
							HIST1H4K	1.649	4.54*E−*11
							HIST1H4J	2.204	4.67*E−*11
							HIST1H3H	1.277	5.05*E−*11
							HIST1H3I	0.574	6.66*E−*11
							HIST3H2BB	0.268	6.82*E−*11
							HIST1H4A	0.474	1.04*E−*10

**rs17801458**	LRP1B (intron 2)		2:141710903	T	C	0.05	HIST1H1B	2.975	1.62*E−*14
							HIST1H2BM	1.369	3.12*E−*14
							HIST1H3B	2.277	1.75*E−*13
							HIST1H4B	1.154	4.90*E−*13
							HIST1H2AB	0.310	8.94*E−*13
							HIST1H3J	1.273	1.28*E−*12
							HIST1H1E	2.439	3.33*E−*12

							HIST1H2AJ	1.711	4.01*E−*12
							HIST1H2AL	0.552	9.35*E−*12
							HIST2H2AB	0.263	1.43*E−*11
							HIST2H3C	0.609	4.10*E−*11
							HIST1H4K	1.649	4.54*E−*11
							HIST1H4J	2.204	4.67*E−*11
							HIST1H3H	1.277	5.05*E−*11
							HIST1H3I	0.574	6.66*E−*11
							HIST3H2BB	0.268	6.82*E−*11
							HIST1H4A	0.474	1.04*E−*10

rs7570091	LRP1B (intron 2)		2:141714131	G	T	0.05	HIST1H2BM	1.297	1.36*E−*13
							HIST1H1B	2.787	1.48*E−*13
							HIST1H3B	2.132	1.34*E−*12
							HIST1H2AL	0.543	4.65*E−*12
							HIST1H4B	1.074	4.79*E−*12
							HIST1H3J	1.201	5.93*E−*12
							HIST1H2AB	0.289	7.28*E−*12
							HIST1H2AJ	1.628	1.12*E−*11
							HIST2H2AB	0.256	1.13*E−*11
							HIST3H2BB	0.267	1.89*E−*11
							HIST1H1E	2.277	2.38*E−*11
							HIST2H3C	0.583	7.77*E−*11
							HIST1H3I	0.545	1.78*E−*10
							HIST1H3H	1.199	2.26*E−*10

**rs11684178**	LRP1B (intron 2)		2:141788340	C	G	0.06	HIST1H2BM	1.090	6.71*E−*12
							HIST1H1B	2.283	2.56*E−*11
							HIST1H3B	1.778	6.72*E−*11
							HIST2H2AB	0.221	1.04*E−*10
							HIST1H3J	1.019	1.10*E−*10
							HIST1H2AL	0.455	1.67*E−*10

**rs6718590**	LRP1B (intron 1)		2:141817609	G	C	0.07	HIST1H2BM	0.968	1.15*E−*10

**rs111875224**	Intergenic		2:202309382	A	C	0.05	HIST1H2AM	0.594	1.24*E−*11
							HIST1H4B	1.023	8.51*E−*11

rs849583	NRP2 (intron 7)		2:205737518	A	G	0.08	HIST1H4A	0.398	8.66*E−*11

rs849578^#^	NRP2 (intron 7)		2:205738798	T	G	0.08	HIST1H4A	0.390	1.69*E−*10

**rs849577**	NRP2 (intron 7)		2:205739705	A	G	0.07	HIST1H4A	0.407	4.48*E−*11

rs849573	NRP2 (intron 8)		2:205741099	T	C	0.08	HIST1H4A	0.407	4.48*E−*11

rs849572	NRP2 (intron 8)		2:205741317	A	G	0.08	HIST1H4A	0.398	8.98*E−*11

rs849567	NRP2 (intron 9)		2:205744540	T	C	0.07	HIST1H4A	0.403	7.11*E−*11

**rs830612**	Intergenic		3:71634179	C	T	0.08	HIST1H4B	0.889	1.28*E−*10

**rs150958961**	Intergenic		4:9006930	C	A	0.1	HIST1H4A	0.341	8.77*E−*11

**rs148619316**	Intergenic		5:14080602	T	C	0.06	HIST1H4B	1.006	4.81*E−*11

rs1992368	Intergenic		5:14092868	A	G	0.06	HIST1H4B	0.974	9.25*E−*11

rs55989629	Intergenic		5:14093354	T	C	0.06	HIST1H4B	0.974	9.25*E−*11

rs11956277	Intergenic		5:14095038	T	C	0.06	HIST1H4B	0.974	9.25*E−*11

rs66689811	Intergenic		5:14095266	T	C	0.06	HIST1H4B	0.974	9.25*E−*11

rs6863067	Intergenic		5:14098914	A	C	0.06	HIST1H4B	0.974	9.25*E−*11

rs10041963	Intergenic		5:14099801	A	G	0.06	HIST1H4B	0.974	9.25*E−*11

rs10072716	Intergenic		5:14101222	T	C	0.06	HIST1H4B	0.974	9.25*E−*11

**rs6861945**	Intergenic		5:53398953	A	G	0.11	HIST1H3J	0.780	2.75*E−*10

**rs61564830**	Intergenic		6:129768950	T	C	0.06	HIST1H3J	1.036	2.62*E−*10

**rs144499291**	UTRN (intron 22)		6:144471095	G	A	0.06	HIST1H4A	0.461	2.88*E−*10

**rs71560740**	Intergenic		7:85896218	C	T	0.06	HIST1H2AJ	1.491	1.48*E−*10
							HIST1H3A	0.228	2.41*E−*10

**rs118052517**	Intergenic		7:153287269	C	T	0.06	HIST1H3J	1.234	8.16*E−*13
							HIST1H4A	0.494	1.89*E−*12
							HIST1H2BM	1.222	2.40*E−*12
							HIST1H2AJ	1.648	3.64*E−*12
							HIST1H3B	2.052	6.16*E−*12
							HIST1H1B	2.540	1.39*E−*11
							HIST1H1E	2.269	1.78*E−*11
							HIST1H2AB	0.270	1.16*E−*10
							HIST1H4K	1.532	2.14*E−*10
							HIST1H4J	2.039	2.62*E−*10
							HIST1H3I	0.534	2.77*E−*10
							HIST1H3H	1.180	2.95*E−*10

**rs77957383**	Intergenic		9:1715766	T	C	0.07	HIST1H4B	0.894	1.85*E−*10

**rs113936625**	Intergenic		10:111124417	C	G	0.05	HIST1H4B	1.056	2.76*E−*10

**rs75201173**	Intergenic	CTCF	11:1062030	C	A	0.07	HIST1H2BM	1.092	1.45*E−*11
							HIST2H3C	0.529	1.22*E−*10
							HIST1H1B	2.227	1.75*E−*10
							HIST1H3B	1.766	1.94*E−*10

rs11245920	Intergenic	CTCF	11:1062519	C	T	0.07	HIST1H2BM	1.092	1.45*E−*11
							HIST2H3C	0.529	1.22*E−*10
							HIST1H1B	2.227	1.75*E−*10
							HIST1H3B	1.766	1.94*E−*10

rs111452409	Intergenic		11:1064929	G	A	0.07	HIST1H2BM	1.092	1.45*E−*11
							HIST2H3C	0.529	1.22*E−*10
							HIST1H1B	2.227	1.75*E−*10
							HIST1H3B	1.766	1.94*E−*10

**rs113659777**	MUC2 (intron 2)		11:1088149	A	G	0.07	HIST1H2BM	1.101	2.89*E−*11
							HIST1H4B	0.951	8.17*E−*11

**rs184239416**	MUC2 (intron 2)		11:1088154	G	A	0.06	HIST1H2BM	1.262	1.56*E−*12
							HIST1H4B	1.077	9.13*E−*12
							HIST1H1B	2.566	2.84*E−*11
							HIST1H3J	1.174	3.88*E−*11
							HIST1H3B	1.979	1.17*E−*10
							HIST1H1E	2.206	2.06*E−*10
							HIST3H2BB	0.257	2.37*E−*10

**rs41392245**	MUC2 (intron 2)		11:1089095	T	C	0.07	HIST1H2BM	1.068	4.19*E−*11
							HIST2H3C	0.528	1.34*E−*10
							HIST1H3B	1.761	2.23*E−*10
							HIST1H1B	2.201	2.90*E−*10

**rs11033879**	MMP26 (intron 1)		11:4786997	G	A	0.05	HIST1H2AJ	1.711	2.53*E−*13
							HIST1H1E	2.374	9.71*E−*13
							HIST1H3B	2.102	9.80*E−*13
							HIST2H2AB	0.263	1.12*E−*12
							HIST1H1B	2.635	1.18*E−*12
							HIST1H4A	0.490	1.62*E−*12
							HIST1H2BM	1.195	4.53*E−*12
							HIST2H3C	0.594	1.15*E−*11
							HIST1H3J	1.148	2.08*E−*11
							HIST1H4K	1.593	2.16*E−*11
							HIST1H2AB	0.275	3.14*E−*11
							HIST1H4J	2.095	4.85*E−*11
							HIST1H3I	0.544	7.46*E−*11
							HIST1H3H	1.190	1.28*E−*10
							HIST1H2AH	0.793	2.70*E−*10

**rs80143982**	Intergenic		11:110990257	A	G	0.05	HIST1H4A	0.482	2.65*E−*10

**rs75349221**	Intergenic		14:44176974	T	A	0.05	HIST1H4A	0.471	2.75*E−*10

**rs72846811**	RBFOX3 (intron 3)		17:79231229	A	G	0.06	HIST1H4A	0.489	1.90*E−*11

**rs183898825**	TAF4B (intron 9)		18:26303160	C	A	0.05	HIST1H4A	0.466	2.60*E−*10

**rs57185274**	CACNA1A (intron 3)		19:13406972	A	T	0.05	HIST1H4B	1.034	2.04*E−*10

^a^SNP in bold indicates the representative variant in each linkage disequilibrium block. SNP with superscript “#” was associated with autism in a Chinese population [15]. ^b^The effects were predicted using Ensembl Variant Effect Predictor program [11]. OC: open chromatin region; CTCF: CTCF binding site. ^c^Chromosome: base pair position from GRCh38.p2. ^d^A1: minor allele; A2: major allele. SNP: single-nucleotide polymorphism; MAF: minor allele frequency.

**Table 2:** Associations of the identified eQTLs with expression of histone and nonhistone genes all over the human genome.

eQTL	Gene	*P*	Gene	*P*	Gene	*P*	Gene	*P*
rs10919229	HIST1H4B (6)	2.51*E−*10	HIST1H3J (6)	3.40*E−*08	HIST1H3H (6)	2.02*E−*07	SASH3 (X)	2.89*E−*06
	HIST1H2BM (6)	4.38*E−*09	HIST1H3B (6)	4.64*E−*08	HIST1H1E (6)	2.50*E−*07	HIST1H2AH (6)	4.61*E−*06
	HIST1H1B (6)	1.81*E−*08	RPPH1 (14)	5.82*E−*08	HIST1H2AB (6)	2.54*E−*07		
	SCARNA13 (14)	3.38*E−*08	SCARNA12 (12)	1.22*E−*07	HIST1H2AM (6)	9.76*E−*07		

rs191508159	SNORA53 (12)	4.38*E−*13	SCARNA2 (1)	5.28*E−*08	HIST2H3C (1)	1.41*E−*07	HIST1H3H (6)	5.46*E−*07
	HIST1H4A (6)	3.41*E−*11	HIST1H1E (6)	7.02*E−*08	HIST1H2AL (6)	1.99*E−*07	G3BP2 (4)	5.51*E−*07
	HIST1H2AJ (6)	5.72*E−*09	HIST2H2AB (1)	7.35*E−*08	HIST1H1B (6)	2.67*E−*07	HIST1H2AH (6)	3.21*E−*06
	HIST1H3J (6)	3.33*E−*08	HIST1H2AB (6)	1.02*E−*07	HIST1H4K (6)	2.99*E−*07	HIST1H3A (6)	4.06*E−*06
	HIST1H4J (6)	4.63*E−*08	HIST1H3B (6)	1.14*E−*07	HIST1H3I (6)	5.12*E−*07	HIST1H2BM (6)	4.73*E−*06

rs13432631	HIST1H4B (6)	1.78*E−*11	HIST1H3B (6)	1.13*E−*08	HIST1H2AJ (6)	1.86*E−*07	HIST1H2AH (6)	6.84*E−*07
	SCARNA12 (12)	1.70*E−*10	HIST1H2AB (6)	2.83*E−*08	HIST3H2BB (1)	1.91*E−*07	RPPH1 (14)	7.69*E−*07
	HIST1H2BM (6)	1.95*E−*09	HIST1H3I (6)	1.01*E−*07	HIST1H3H (6)	2.56*E−*07	HIST1H3A (6)	2.10*E−*06
	HIST1H1B (6)	3.33*E−*09	SCARNA13 (14)	1.23*E−*07	HIST1H4K (6)	3.45*E−*07	HIST1H4J (6)	2.48*E−*06
	HIST1H3J (6)	1.11*E−*08	HIST1H1E (6)	1.74*E−*07	HIST2H2AB (1)	6.28*E−*07	HIST1H4A (6)	2.81*E−*06

rs189340111	HIST1H4B (6)	2.99*E−*12	HIST1H3I (6)	1.73*E−*09	HIST1H4J (6)	3.30*E−*08	SCARNA2 (1)	4.27*E−*07
	HIST1H1B (6)	2.91*E−*11	HIST2H2AB (1)	1.78*E−*09	HIST1H3A (6)	3.60*E−*08	PIGF (2)	6.15*E−*07
	HIST1H1E (6)	6.90*E−*11	HIST1H2AJ (6)	2.58*E−*09	HIST1H4A (6)	7.70*E−*08	SCARNA13 (14)	1.55*E−*06
	HIST1H3J (6)	8.17*E−*11	HIST1H3H (6)	3.93*E−*09	HIST1H2AM (6)	1.00*E−*07	RPPH1 (14)	1.91*E−*06
	HIST1H2AH (6)	2.34*E−*10	HIST1H4K (6)	9.71*E−*09	SCARNA12 (12)	1.05*E−*07	HIST1H2AL (6)	2.40*E−*06
	HIST1H2BM (6)	2.58*E−*10	HIST1H2AB (6)	1.81*E−*08	SCARNA7 (3)	1.81*E−*07		
	HIST1H3B (6)	4.62*E−*10	HIST3H2BB (1)	3.14*E−*08	HIST2H3C (1)	2.90*E−*07		

rs75372391	HIST1H2BM (6)	6.19*E−*12	HIST1H3I (6)	5.56*E−*09	HIST1H4A (6)	3.57*E−*08	HIST1H2AG (6)	1.77*E−*07
	HIST1H4B (6)	1.39*E−*11	HIST1H2AJ (6)	6.33*E−*09	HIST1H2AH (6)	5.13*E−*08	HIST1H2AK (6)	6.45*E−*07
	HIST1H1B (6)	2.92*E−*11	HIST1H2AB (6)	7.88*E−*09	HIST1H2AM (6)	5.45*E−*08	SCARNA7 (3)	1.95*E−*06
	RPPH1 (14)	1.53*E−*10	HIST3H2BB (1)	9.94*E−*09	HIST1H4J (6)	5.90*E−*08	HIST1H3A (6)	2.31*E−*06
	HIST1H3B (6)	2.00*E−*10	HIST1H3H (6)	1.10*E−*08	HIST2H3C (1)	9.35*E−*08	SCARNA2 (1)	2.85*E−*06
	HIST1H1E (6)	6.26*E−*10	HIST2H2AB (1)	2.33*E−*08	SCARNA12 (12)	9.42*E−*08		
	HIST1H3J (6)	1.15*E−*09	HIST1H4K (6)	3.21*E−*08	HIST1H2AL (6)	1.09*E−*07		

rs72853591	HIST1H4B (6)	9.68*E−*11	SCARNA13 (14)	1.03*E−*08	SCARNA12 (12)	2.12*E−*07	HIST1H2AH (6)	2.69*E−*06
	HIST1H1B (6)	5.88*E−*10	HIST1H3J (6)	1.17*E−*08	HIST2H2AB (1)	4.57*E−*07	HIST1H2AL (6)	2.72*E−*06
	HIST1H2BM (6)	6.02*E−*10	HIST1H1E (6)	1.88*E−*08	HIST1H4A (6)	1.16*E−*06	HIST1H4J (6)	3.52*E−*06
	RPPH1 (14)	1.96*E−*09	HIST1H3H (6)	1.56*E−*07	HIST1H4K (6)	1.57*E−*06	HIST1H3A (6)	4.33*E−*06
	HIST1H3B (6)	4.11*E−*09	HIST1H2AJ (6)	1.74*E−*07	SNORD17 (20)	1.66*E−*06	HIST2H3C (1)	4.67*E−*06
	HIST1H3I (6)	8.43*E−*09	HIST1H2AB (6)	1.84*E−*07	SCARNA2 (1)	2.24*E−*06		

rs17801458	RPPH1 (14)	1.31*E−*15	HIST1H2AL (6)	9.35*E−*12	SCARNA2 (1)	9.35*E−*10	HIST1H2AK (6)	1.75*E−*06
	HIST1H1B (6)	1.62*E−*14	HIST2H2AB (1)	1.43*E−*11	HIST1H3A (6)	5.23*E−*09	VGLL4 (3)	1.88*E−*06
	HIST1H2BM (6)	3.12*E−*14	HIST2H3C (1)	4.10*E−*11	HIST1H2AG (6)	6.96*E−*09	SNORA53 (12)	2.09*E−*06
	HIST1H3B (6)	1.75*E−*13	HIST1H4K (6)	4.54*E−*11	HIST1H2AH (6)	1.34*E−*08	WASF2 (1)	3.49*E−*06
	HIST1H4B (6)	4.90*E−*13	HIST1H4J (6)	4.67*E−*11	SNORD17 (20)	2.36*E−*08	UBE2R2 (9)	4.47*E−*06
	HIST1H2AB (6)	8.94*E−*13	HIST1H3H (6)	5.05*E−*11	SCARNA12 (12)	2.60*E−*08		
	HIST1H3J (6)	1.28*E−*12	HIST1H3I (6)	6.66*E−*11	SCARNA7 (3)	1.91*E−*07		
	HIST1H1E (6)	3.33*E−*12	HIST3H2BB (1)	6.82*E−*11	SCARNA13 (14)	4.53*E−*07		
	HIST1H2AJ (6)	4.01*E−*12	HIST1H4A (6)	1.04*E−*10	HIST1H2AM (6)	1.16*E−*06		

rs11684178	HIST1H2BM (6)	6.71*E−*12	HIST1H1E (6)	3.59*E−*10	HIST1H3A (6)	2.78*E−*09	NOL11 (17)	4.61*E−*07
	RPPH1 (14)	7.92*E−*12	HIST1H4J (6)	3.64*E−*10	HIST2H3C (1)	3.55*E−*09	SCARNA12 (12)	8.16*E−*07
	HIST1H1B (6)	2.56*E−*11	HIST1H4B (6)	3.69*E−*10	HIST3H2BB (1)	9.84*E−*09	SCARNA13 (14)	1.09*E−*06
	HIST1H3B (6)	6.72*E−*11	HIST1H2AB (6)	4.40*E−*10	HIST1H4A (6)	2.03*E−*08	DDX17 (22)	1.32*E−*06
	HIST2H2AB (1)	1.04*E−*10	HIST1H2AH (6)	7.01*E−*10	HIST1H4K (6)	3.26*E−*08	MRPS24 (7)	3.22*E−*06
	HIST1H3J (6)	1.10*E−*10	HIST1H2AJ (6)	8.58*E−*10	SCARNA7 (3)	3.37*E−*08	FASTKD2 (2)	3.42*E−*06
	HIST1H2AL (6)	1.67*E−*10	HIST1H3H (6)	1.56*E−*09	HIST1H2AG (6)	3.80*E−*08	SNORD17 (20)	3.99*E−*06
	HIST1H3I (6)	3.41*E−*10	SCARNA2 (1)	2.47*E−*09	HIST1H2AK (6)	7.75*E−*08	EFTUD2 (17)	4.04*E−*06

rs6718590	HIST1H2BM (6)	1.15*E−*10	HIST1H1E (6)	2.68*E−*09	HIST1H3H (6)	1.57*E−*08	NOL11 (17)	4.12*E−*07
	RPPH1 (14)	1.92*E−*10	HIST1H3I (6)	3.57*E−*09	HIST1H3A (6)	1.98*E−*08	SCARNA12 (12)	1.10*E−*06
	HIST1H1B (6)	3.63*E−*10	HIST1H4B (6)	4.90*E−*09	SCARNA2 (1)	2.30*E−*08	APPBP2 (17)	1.77*E−*06
	HIST1H2AL (6)	4.03*E−*10	HIST1H2AB (6)	6.23*E−*09	HIST1H2AG (6)	8.36*E−*08	ATP5A1 (18)	3.68*E−*06
	HIST2H2AB (1)	5.83*E−*10	HIST1H2AH (6)	6.89*E−*09	HIST1H4A (6)	1.92*E−*07	PITPNB (22)	3.92*E−*06
	HIST1H3J (6)	9.78*E−*10	HIST1H4J (6)	9.48*E−*09	SCARNA7 (3)	2.51*E−*07		
	HIST3H2BB (1)	1.30*E−*09	HIST2H3C (1)	1.01*E−*08	HIST1H4K (6)	3.19*E−*07		
	HIST1H3B (6)	1.74*E−*09	HIST1H2AJ (6)	1.10*E−*08	HIST1H2AK (6)	3.22*E−*07		

rs111875224	HIST1H2AM (6)	1.24*E−*11	SCARNA7 (3)	2.79*E−*08	HIST1H3H (6)	2.63*E−*07	GTF2E2 (8)	2.28*E−*06
	HIST1H4B (6)	8.51*E−*11	HIST3H2BB (1)	3.71*E−*08	SCARNA2 (1)	3.89*E−*07	C18orf32 (18)	2.68*E−*06
	HIST1H1B (6)	7.63*E−*10	HIST1H3J (6)	9.07*E−*08	HIST1H2AJ (6)	4.51*E−*07	CD63 (12)	2.80*E−*06
	HIST1H1E (6)	1.11*E−*09	SCARNA12 (12)	1.02*E−*07	SCARNA13 (14)	4.99*E−*07	SERINC1 (6)	3.24*E−*06
	HIST1H2BM (6)	1.41*E−*09	HIST1H3I (6)	1.14*E−*07	HIST1H3A (6)	9.59*E−*07		
	HIST1H3B (6)	1.41*E−*08	HIST1H2AB (6)	1.32*E−*07	HIST1H2AL (6)	1.60*E−*06		
	HIST1H2AH (6)	2.27*E−*08	HIST2H2AB (1)	1.38*E−*07	RPPH1 (14)	1.87*E−*06		

rs849577	HIST1H4A (6)	4.48*E−*11	HIST1H1E (6)	3.42*E−*08	HIST1H3I (6)	4.33*E−*07	HIST1H3A (6)	2.03*E−*06
	SNORA53 (12)	3.16*E−*10	HIST2H2AB (1)	4.00*E−*08	HIST1H1B (6)	5.65*E−*07	HIST1H3H (6)	2.06*E−*06
	HIST1H4J (6)	8.79*E−*10	HIST1H3B (6)	8.53*E−*08	HIST1H3J (6)	6.70*E−*07	RPPH1 (14)	3.22*E−*06
	SCARNA2 (1)	1.23*E−*09	HIST1H2AL (6)	1.35*E−*07	HIST1H2AH (6)	6.75*E−*07	HIST1H2BM (6)	4.42*E−*06
	HIST1H4K (6)	2.46*E−*09	HIST2H3C (1)	2.09*E−*07	SCARNA7 (3)	1.71*E−*06		
	HIST1H2AJ (6)	1.12*E−*08	HIST1H2AB (6)	3.52*E−*07	SNORD17 (20)	1.77*E−*06		

rs830612	HIST1H4B (6)	1.28*E−*10	HIST1H3B (6)	1.16*E−*08	SNORD17 (20)	1.52*E−*07	RPPH1 (14)	6.10*E−*07
	HIST1H1B (6)	6.19*E−*10	HIST1H3H (6)	1.67*E−*08	SASH3 (X)	1.74*E−*07	HIST1H2AJ (6)	6.67*E−*07
	HIST1H2BM (6)	2.66*E−*09	HIST1H1E (6)	2.00*E−*08	HIST1H2AH (6)	2.54*E−*07	SCARNA7 (3)	1.56*E−*06
	HIST1H3J (6)	1.05*E−*08	HIST1H3I (6)	4.17*E−*08	HIST1H3A (6)	3.80*E−*07	HIST1H2AB (6)	3.15*E−*06

rs150958961	HIST1H4A (6)	8.77*E−*11	HIST1H1B (6)	4.03*E−*09	HIST1H4K (6)	3.84*E−*08	HIST2H2AB (1)	4.70*E−*07
	HIST1H3J (6)	3.41*E−*10	SCARNA2 (1)	6.96*E−*09	HIST1H3H (6)	4.81*E−*08	SCARNA7 (3)	5.49*E−*07
	HIST1H2AJ (6)	3.78*E−*10	HIST1H2AH (6)	1.72*E−*08	HIST1H3I (6)	1.01*E−*07	HIST1H3A (6)	1.04*E−*06
	HIST1H2AB (6)	3.81*E−*10	HIST1H2AL (6)	1.86*E−*08	HIST1H4B (6)	1.38*E−*07	SNORA57 (11)	1.66*E−*06
	HIST1H3B (6)	4.54*E−*10	HIST1H2BM (6)	2.30*E−*08	HIST3H2BB (1)	1.62*E−*07	HIST1H2AG (6)	2.46*E−*06
	RPPH1 (14)	7.11*E−*10	HIST1H4J (6)	2.31*E−*08	SNORA53 (12)	4.22*E−*07		
	HIST1H1E (6)	1.80*E−*09	HIST2H3C (1)	2.57*E−*08	SNORD17 (20)	4.60*E−*07		

rs148619316	HIST1H4B (6)	4.81*E−*11	HIST1H3H (6)	6.81*E−*08	HIST1H2AJ (6)	1.23*E−*06	PPM1G (2)	2.97*E−*06
	RPPH1 (14)	3.50*E−*09	HIST1H1E (6)	1.11*E−*07	HIST1H3I (6)	1.33*E−*06	THRAP3 (1)	3.14*E−*06
	HIST1H1B (6)	4.92*E−*09	HIST1H2AH (6)	1.66*E−*07	SNORD17 (20)	1.52*E−*06	SUPT5H (19)	3.91*E−*06
	HIST1H3J (6)	5.46*E−*09	DDX28 (16)	5.22*E−*07	SCARNA12 (12)	1.68*E−*06	ZNF207 (17)	4.48*E−*06
	HIST1H3B (6)	9.95*E−*09	HIST1H2AB (6)	6.80*E−*07	HIST1H2AK (6)	1.69*E−*06		
	HIST1H2BM (6)	1.70*E−*08	HIST2H2AB (1)	1.14*E−*06	C20orf11 (20)	2.61*E−*06		
	SASH3 (X)	3.00*E−*08	HIST3H2BB (1)	1.22*E−*06	SCARNA7 (3)	2.67*E−*06		

rs6861945	HIST1H3J (6)	2.75*E−*10	HIST1H4J (6)	1.67*E−*08	HIST1H3I (6)	7.68*E−*08	HIST1H2AK (6)	1.18*E−*06
	HIST1H3B (6)	4.30*E−*09	HIST1H1E (6)	2.30*E−*08	HIST1H2AB (6)	1.38*E−*07	HIST2H2AB (1)	1.32*E−*06
	HIST1H4B (6)	4.44*E−*09	HIST1H4K (6)	3.40*E−*08	SCARNA2 (1)	1.55*E−*07	SCARNA7 (3)	2.25*E−*06
	HIST1H2BM (6)	7.98*E−*09	HIST1H2AH (6)	3.78*E−*08	HIST1H4A (6)	1.55*E−*07	SNORD17 (20)	2.57*E−*06
	HIST1H1B (6)	1.04*E−*08	HIST1H3H (6)	4.52*E−*08	SASH3 (X)	4.33*E−*07	HIST1H2AL (6)	4.34*E−*06
	RPPH1 (14)	1.11*E−*08	HIST1H2AJ (6)	5.09*E−*08	HIST1H3A (6)	8.63*E−*07		

rs61564830	HIST1H3J (6)	2.62*E−*10	SCARNA2 (1)	5.85*E−*09	HIST1H3I (6)	3.37*E−*08	HIST1H2AB (6)	2.54*E−*06
	HIST1H2AH (6)	8.25*E−*10	HIST1H1B (6)	6.35*E−*09	HIST1H2BM (6)	3.45*E−*08	HIST1H2AL (6)	2.85*E−*06
	HIST1H1E (6)	9.74*E−*10	HIST1H2AJ (6)	1.00*E−*08	SCARNA13 (14)	1.68*E−*07	UBE2R2 (9)	4.53*E−*06
	HIST1H3B (6)	1.50*E−*09	SNORD17 (20)	1.23*E−*08	RPPH1 (14)	3.47*E−*07		
	HIST1H3H (6)	1.58*E−*09	SCARNA7 (3)	2.46*E−*08	HIST2H2AB (1)	2.23*E−*06		
	HIST1H4B (6)	2.05*E−*09	HIST1H4A (6)	2.75*E−*08	MRPS31 (13)	2.36*E−*06		

rs144499291	HIST1H4A (6)	2.88*E−*10	HIST1H2AK (6)	4.16*E−*09	HIST1H4K (6)	9.65*E−*08	FAM122A (9)	2.05*E−*06
	HIST1H1B (6)	4.57*E−*10	HIST1H3H (6)	5.46*E−*09	KPNB1 (17)	1.48*E−*07	RPPH1 (14)	3.45*E−*06
	HIST1H2AJ (6)	7.99*E−*10	HIST1H3I (6)	1.06*E−*08	SNORD17 (20)	1.49*E−*07	HIST3H2BB (1)	4.06*E−*06
	HIST1H1E (6)	9.23*E−*10	HIST1H2AB (6)	1.12*E−*08	HIST2H3C (1)	2.10*E−*07		
	HIST1H2BM (6)	1.15*E−*09	HIST2H2AB (1)	3.99*E−*08	HIST1H4J (6)	2.73*E−*07		
	HIST1H3J (6)	2.45*E−*09	HIST1H4B (6)	4.58*E−*08	SCARNA7 (3)	2.83*E−*07		
	HIST1H3B (6)	2.85*E−*09	SCARNA2 (1)	5.61*E−*08	HIST1H2AL (6)	4.25*E−*07		
	HIST1H2AH (6)	4.05*E−*09	SNORA53 (12)	9.55*E−*08	HIST1H3A (6)	5.03*E−*07		

rs71560740	HIST1H2AJ (6)	1.48*E−*10	HIST1H3I (6)	6.75*E−*09	RPPH1 (14)	1.71*E−*08	HIST1H2AH (6)	4.57*E−*07
	HIST1H3A (6)	2.41*E−*10	HIST2H2AB (1)	9.23*E−*09	SNORA53 (12)	2.32*E−*08	HIST1H3H (6)	9.55*E−*07
	HIST1H2AB (6)	8.54*E−*10	HIST1H4J (6)	9.80*E−*09	HIST1H3J (6)	2.54*E−*08	TDRD12 (19)	3.26*E−*06
	HIST2H3C (1)	1.32*E−*09	HIST1H2BM (6)	1.13*E−*08	HIST1H1E (6)	3.18*E−*08	HIST1H2AM (6)	3.74*E−*06
	HIST1H3B (6)	3.46*E−*09	HIST1H4A (6)	1.27*E−*08	HIST1H2AL (6)	7.54*E−*08	HIST1H4B (6)	3.78*E−*06
	HIST1H1B (6)	6.55*E−*09	HIST1H4K (6)	1.29*E−*08	SCARNA2 (1)	2.35*E−*07		

rs118052517	HIST1H3J (6)	8.16*E−*13	HIST1H4K (6)	2.14*E−*10	HIST1H4B (6)	2.72*E−*09	HIST3H2BB (1)	3.49*E−*08
	HIST1H4A (6)	1.89*E−*12	SCARNA2 (1)	2.30*E−*10	HIST1H2AH (6)	2.81*E−*09	LOXL4 (10)	1.35*E−*06
	HIST1H2BM (6)	2.40*E−*12	HIST1H4J (6)	2.62*E−*10	HIST2H3C (1)	6.12*E−*09	KRT8 (12)	1.53*E−*06
	HIST1H2AJ (6)	3.64*E−*12	HIST1H3I (6)	2.77*E−*10	SNORA53 (12)	8.62*E−*09	HIST1H2AM (6)	1.63*E−*06
	HIST1H3B (6)	6.16*E−*12	HIST1H3H (6)	2.95*E−*10	HIST1H3A (6)	1.04*E−*08	TRNP1 (1)	1.82*E−*06
	HIST1H1B (6)	1.39*E−*11	RPPH1 (14)	1.53*E−*09	HIST1H2AK (6)	1.27*E−*08	SCUBE2 (11)	4.20*E−*06
	HIST1H1E (6)	1.78*E−*11	HIST1H2AL (6)	1.73*E−*09	SNORD17 (20)	1.61*E−*08		
	HIST1H2AB (6)	1.16*E−*10	HIST2H2AB (1)	2.34*E−*09	SCARNA7 (3)	3.31*E−*08		

rs77957383	HIST1H4B (6)	1.85*E−*10	HIST3H2BB (1)	4.38*E−*09	SCARNA12 (12)	1.82*E−*07	CDC5L (6)	3.27*E−*06
	RPPH1 (14)	3.19*E−*10	HIST1H1E (6)	2.17*E−*08	SCARNA2 (1)	2.84*E−*07	HIST2H3C (1)	4.43*E−*06
	HIST1H2BM (6)	4.46*E−*10	HIST2H2AB (1)	2.20*E−*08	HIST1H3H (6)	7.97*E−*07		
	HIST1H3B (6)	1.04*E−*09	HIST1H2AJ (6)	2.95*E−*08	HIST1H2AK (6)	1.03*E−*06		
	HIST1H1B (6)	1.38*E−*09	SCARNA7 (3)	3.95*E−*08	HIST1H4A (6)	1.61*E−*06		
	HIST1H3J (6)	3.57*E−*09	HIST1H3I (6)	4.73*E−*08	SCARNA13 (14)	2.67*E−*06		
	HIST1H2AB (6)	3.98*E−*09	HIST1H2AH (6)	1.49*E−*07	HIST1H2AL (6)	3.22*E−*06		

rs113936625	HIST1H4B (6)	2.76*E−*10	SCARNA13 (14)	1.12*E−*07	HIST1H3H (6)	3.64*E−*07	RPPH1 (14)	2.97*E−*06
	HIST1H2BM (6)	2.29*E−*09	SCARNA12 (12)	1.20*E−*07	HIST1H2AJ (6)	9.36*E−*07	HIST1H2AL (6)	3.00*E−*06
	HIST1H1B (6)	8.82*E−*09	HIST1H3B (6)	1.36*E−*07	HIST2H2AB (1)	1.25*E−*06	HIST1H3A (6)	3.44*E−*06
	HIST1H2AM (6)	1.42*E−*08	HIST1H2AB (6)	1.79*E−*07	HIST1H3I (6)	1.41*E−*06	SNORD17 (20)	3.63*E−*06
	HIST1H3J (6)	3.32*E−*08	HIST1H1E (6)	2.11*E−*07	HIST1H2AH (6)	2.45*E−*06		

rs75201173	HIST1H2BM (6)	1.45*E−*11	HIST1H2AK (6)	2.05*E−*09	STYX (14)	4.33*E−*07	HMHA1 (19)	2.49*E−*06
	RPPH1 (14)	6.79*E−*11	HIST1H2AB (6)	3.62*E−*09	PRKAR1A (17)	4.54*E−*07	OSBPL11 (3)	2.57*E−*06
	HIST2H3C (1)	1.22*E−*10	SCARNA2 (1)	1.15*E−*08	SMAD2 (18)	5.85*E−*07	FBXL12 (19)	2.73*E−*06
	HIST1H1B (6)	1.75*E−*10	HIST1H2AL (6)	1.56*E−*08	EFTUD2 (17)	6.59*E−*07	FNBP1 (9)	2.92*E−*06
	HIST1H3B (6)	1.94*E−*10	HIST2H2AB (1)	3.33*E−*08	ZFP91 (11)	8.19*E−*07	SLC44A2 (19)	3.26*E−*06
	HIST1H4B (6)	3.04*E−*10	HIST1H3I (6)	5.13*E−*08	GBF1 (10)	1.03*E−*06	SCARNA12 (12)	3.62*E−*06
	HIST1H3H (6)	6.76*E−*10	HIST1H2AH (6)	5.81*E−*08	APBA3 (19)	1.15*E−*06	SCARNA13 (14)	4.24*E−*06
	HIST1H2AJ (6)	6.95*E−*10	HIST1H2AG (6)	7.13*E−*08	COX15 (10)	1.90*E−*06	ACLY (17)	4.49*E−*06
	SCARNA7 (3)	1.45*E−*09	HIST1H4K (6)	1.46*E−*07	SERBP1 (1)	2.00*E−*06		
	HIST1H2AM (6)	1.61*E−*09	HIST3H2BB (1)	1.64*E−*07	HIST1H2BN (6)	2.09*E−*06		
	HIST1H1E (6)	1.79*E−*09	HIST1H4A (6)	3.81*E−*07	HIST1H3A (6)	2.19*E−*06		
	HIST1H3J (6)	1.92*E−*09	HIST1H4J (6)	4.30*E−*07	HIST1H2BO (6)	2.22*E−*06		

rs113659777	HIST1H2BM (6)	2.89*E−*11	HIST1H1E (6)	1.72*E−*09	SNORD17 (20)	1.14*E−*07	SCARNA12 (12)	1.62*E−*06
	RPPH1 (14)	7.27*E−*11	HIST1H4K (6)	3.31*E−*09	HIST3H2BB (1)	1.27*E−*07	HTATSF1 (X)	2.97*E−*06
	HIST1H4B (6)	8.17*E−*11	HIST1H2AJ (6)	5.31*E−*09	HIST1H2AK (6)	1.40*E−*07	AGGF1 (5)	3.52*E−*06
	HIST1H1B (6)	3.27*E−*10	HIST1H2AB (6)	7.80*E−*09	SCARNA13 (14)	2.84*E−*07	MTFMT (15)	3.89*E−*06
	HIST1H3B (6)	6.97*E−*10	HIST1H2AL (6)	1.05*E−*08	HIST1H4A (6)	3.39*E−*07		
	HIST2H2AB (1)	9.75*E−*10	SCARNA2 (1)	1.72*E−*08	HIST1H3I (6)	3.63*E−*07		
	HIST1H2AM (6)	9.86*E−*10	HIST1H4J (6)	4.06*E−*08	HIST1H2AG (6)	4.70*E−*07		
	HIST1H3J (6)	1.08*E−*09	HIST1H2AH (6)	9.03*E−*08	HIST1H3A (6)	4.83*E−*07		
	HIST1H3H (6)	1.37*E−*09	HIST2H3C (1)	9.54*E−*08	SCARNA7 (3)	1.13*E−*06		

rs184239416	HIST1H2BM (6)	1.56*E−*12	HIST2H2AB (1)	2.30*E−*09	SCARNA12 (12)	9.43*E−*08	HIST2H3C (1)	1.42*E−*06
	RPPH1 (14)	2.76*E−*12	HIST1H3H (6)	2.98*E−*09	HIST1H4A (6)	9.92*E−*08	GNAI3 (1)	1.60*E−*06
	HIST1H4B (6)	9.13*E−*12	HIST1H2AL (6)	3.09*E−*09	SCARNA13 (14)	1.11*E−*07	MAPRE1 (20)	1.73*E−*06
	HIST1H1B (6)	2.84*E−*11	HIST1H2AJ (6)	3.83*E−*09	HIST1H3I (6)	1.46*E−*07	RIOK3 (18)	2.38*E−*06
	HIST1H3J (6)	3.88*E−*11	HIST1H2AM (6)	8.62*E−*09	CAPZA1 (1)	1.84*E−*07	HIST1H2AK (6)	2.55*E−*06
	HIST1H3B (6)	1.17*E−*10	HIST1H2AB (6)	1.50*E−*08	HIST1H2AG (6)	2.41*E−*07	AGGF1 (5)	2.75*E−*06
	HIST1H1E (6)	2.06*E−*10	SCARNA2 (1)	3.83*E−*08	HIST1H3A (6)	2.62*E−*07	ARL6IP6 (2)	3.00*E−*06
	HIST3H2BB (1)	2.37*E−*10	HIST1H2AH (6)	6.24*E−*08	HIST1H4K (6)	4.45*E−*07		
	SNORD17 (20)	1.19*E−*09	SCARNA7 (3)	6.81*E−*08	HIST1H4J (6)	4.76*E−*07		

rs41392245	HIST1H2BM (6)	4.19*E−*11	HIST1H1E (6)	3.18*E−*09	HIST1H4K (6)	2.14*E−*07	HMHA1 (19)	2.79*E−*06
	RPPH1 (14)	1.07*E−*10	HIST1H2AB (6)	4.10*E−*09	PRKAR1A (17)	3.46*E−*07	FBXL12 (19)	3.05*E−*06
	HIST2H3C (1)	1.34*E−*10	HIST1H2AK (6)	1.08*E−*08	ZFP91 (11)	3.49*E−*07	SNORD17 (20)	3.06*E−*06
	HIST1H3B (6)	2.23*E−*10	SCARNA2 (1)	1.47*E−*08	SCARNA12 (12)	4.21*E−*07	APBA3 (19)	3.07*E−*06
	HIST1H1B (6)	2.90*E−*10	HIST1H2AL (6)	1.89*E−*08	STYX (14)	7.62*E−*07	HIST1H2BN (6)	3.26*E−*06
	HIST1H4B (6)	3.59*E−*10	HIST2H2AB (1)	2.27*E−*08	HIST1H4J (6)	7.71*E−*07	RDH5 (12)	3.79*E−*06
	HIST1H3H (6)	1.08*E−*09	HIST1H2AG (6)	2.99*E−*08	HIST1H4A (6)	9.00*E−*07	ACLY (17)	4.07*E−*06
	HIST1H2AJ (6)	1.10*E−*09	HIST1H2AH (6)	4.46*E−*08	HIST1H2BO (6)	9.37*E−*07	ZNF622 (5)	4.27*E−*06
	SCARNA7 (3)	1.16*E−*09	HIST1H3I (6)	7.59*E−*08	SMAD2 (18)	1.58*E−*06		
	HIST1H2AM (6)	2.39*E−*09	HIST3H2BB (1)	1.42*E−*07	HIST1H3A (6)	1.82*E−*06		
	HIST1H3J (6)	2.76*E−*09	COX15 (10)	1.96*E−*07	EFTUD2 (17)	2.57*E−*06		

rs11033879	SNORA53 (12)	2.29*E−*13	HIST1H3J (6)	2.08*E−*11	HIST1H3A (6)	4.97*E−*10	HIST1H2AG (6)	2.23*E−*07
	HIST1H2AJ (6)	2.53*E−*13	HIST1H4K (6)	2.16*E−*11	RPPH1 (14)	1.18*E−*09	OBFC2B (12)	1.22*E−*06
	HIST1H1E (6)	9.71*E−*13	HIST1H2AB (6)	3.14*E−*11	HIST3H2BB (1)	1.51*E−*09	RUNDC1 (17)	1.82*E−*06
	HIST1H3B (6)	9.80*E−*13	HIST1H4J (6)	4.85*E−*11	HIST1H4B (6)	2.07*E−*09	NHS (X)	1.92*E−*06
	HIST2H2AB (1)	1.12*E−*12	HIST1H3I (6)	7.46*E−*11	HIST1H2AK (6)	6.39*E−*09	SVIL (10)	4.02*E−*06
	HIST1H1B (6)	1.18*E−*12	SCARNA2 (1)	1.10*E−*10	HIST1H2AL (6)	1.64*E−*08		
	HIST1H4A (6)	1.62*E−*12	HIST1H3H (6)	1.28*E−*10	SCARNA7 (3)	3.12*E−*08		
	HIST1H2BM (6)	4.53*E−*12	HIST1H2AH (6)	2.70*E−*10	FN1 (2)	3.67*E−*08		
	HIST2H3C (1)	1.15*E−*11	SNORD17 (20)	4.02*E−*10	HIST1H2AM (6)	1.68*E−*07		

rs80143982	HIST1H4A (6)	2.65*E−*10	NOTCH2NL (1)	2.32*E−*07	HIST1H3I (6)	1.73*E−*06	ZNF207 (17)	4.57*E−*06
	HIST3H2BB (1)	3.31*E−*10	RNF26 (11)	3.73*E−*07	HIST1H2AJ (6)	2.16*E−*06		
	SNORA53 (12)	2.42*E−*08	SCARNA2 (1)	6.10*E−*07	SNORD17 (20)	2.25*E−*06		
	HIST1H1E (6)	3.15*E−*08	HIST2H3C (1)	1.51*E−*06	MRFAP1L1 (4)	4.02*E−*06		

rs75349221	HIST1H4A (6)	2.75*E−*10	HIST1H2BM (6)	8.36*E−*09	HIST1H3H (6)	1.11*E−*07	HIST1H2AB (6)	1.91*E−*06
	HIST1H1E (6)	4.32*E−*10	HIST1H3I (6)	8.42*E−*09	SCARNA2 (1)	1.45*E−*07	NDUFA8 (9)	2.23*E−*06
	HIST1H1B (6)	7.02*E−*10	SNORD17 (20)	1.32*E−*08	HIST1H4K (6)	1.59*E−*07	NDUFS1 (2)	2.90*E−*06
	HIST1H3B (6)	1.26*E−*09	SNORA53 (12)	1.96*E−*08	HIST1H4J (6)	2.64*E−*07	RPPH1 (14)	2.95*E−*06
	HIST1H2AJ (6)	4.14*E−*09	HIST1H4B (6)	2.78*E−*08	UBAP1 (9)	3.35*E−*07	TEX261 (2)	3.35*E−*06
	SCARNA7 (3)	7.10*E−*09	HIST2H2AB (1)	5.63*E−*08	HIST2H3C (1)	1.36*E−*06	RUNDC1 (17)	3.63*E−*06
	HIST1H3J (6)	8.33*E−*09	HIST1H2AH (6)	8.54*E−*08	SCARNA13 (14)	1.61*E−*06	HIST1H2AL (6)	4.38*E−*06

rs72846811	HIST1H4A (6)	1.90*E−*11	HIST1H3I (6)	1.06*E−*07	SCARNA7 (3)	3.49*E−*07	HIST1H3A (6)	2.08*E−*06
	SNORA53 (12)	1.90*E−*10	SCARNA2 (1)	1.22*E−*07	HIST1H3J (6)	5.24*E−*07	HIST1H2BM (6)	2.77*E−*06
	HIST1H1E (6)	8.56*E−*09	HIST1H2AH (6)	2.32*E−*07	HIST2H3C (1)	1.12*E−*06	HIST2H2AB (1)	2.79*E−*06
	HIST1H2AJ (6)	7.92*E−*08	HIST1H3B (6)	2.44*E−*07	HIST1H1B (6)	1.24*E−*06		

rs183898825	SNORA53 (12)	9.65*E−*11	HIST1H4J (6)	2.52*E−*08	HIST1H1E (6)	2.74*E−*07	HIST1H2AH (6)	7.54*E−*07
	HIST1H4A (6)	2.60*E−*10	HIST1H2AJ (6)	4.29*E−*08	HIST2H2AB (1)	4.28*E−*07	SNORA70 (X)	8.38*E−*07
	HIST1H4K (6)	7.93*E−*09	HIST2H3C (1)	8.18*E−*08	HIST1H3B (6)	4.88*E−*07	HIST1H1B (6)	1.58*E−*06
	HIST1H3I (6)	1.64*E−*08	SCARNA7 (3)	1.21*E−*07	HIST1H3H (6)	4.96*E−*07	HIST1H2AB (6)	2.66*E−*06
	SCARNA2 (1)	1.69*E−*08	SNORD17 (20)	1.78*E−*07	HIST1H3A (6)	5.65*E−*07	HIST1H3J (6)	3.18*E−*06

rs57185274	HIST1H4B (6)	2.04*E−*10	HIST1H3J (6)	4.54*E−*08	HIST1H2AB (6)	5.47*E−*07	EPCAM (2)	2.65*E−*06
	HIST1H2BM (6)	3.32*E−*09	HIST1H3B (6)	7.51*E−*08	HIST1H3H (6)	6.20*E−*07	HIST2H3C (1)	2.79*E−*06
	HIST1H2AM (6)	5.05*E−*09	SCARNA12 (12)	2.88*E−*07	HIST1H2AJ (6)	7.13*E−*07	FNBP1 (9)	3.03*E−*06
	HIST1H1B (6)	7.66*E−*09	SCARNA13 (14)	4.61*E−*07	HIST2H2AB (1)	9.68*E−*07	HIST3H2BB (1)	3.24*E−*06
	HIST1H1E (6)	2.02*E−*08	HIST1H3I (6)	5.12*E−*07	HIST1H2AH (6)	1.69*E−*06	ZNF524 (19)	4.57*E−*06

Figure in parentheses indicates chromosome number.
