# Supplementary material for: Common Expression Quantitative Trait Loci Shared by Histone Genes
Source: Int J Genomics. 2017 Aug 27;2017:6202567. doi: 10.1155/2017/6202567 (PMC5591967; doi:10.1155/2017/6202567)
Supplement: Supplementary file 1 — Table S1. Functional capability of eQTLs identified for histones using RegulomeDB. [file 6202567.f1.docx]

TableS1. Functional capability of eQTLs identified for histones using RegulomeDB.

| eQTL | Functions | Score^a^ |
| --- | --- | --- |
| rs79335804 | Motifs\|Footprinting\|Mcf7\|Klf4, Motifs\|Footprinting\|Huh75\|Klf4, Motifs\|Footprinting\|Htr8\|Klf4, Motifs\|Footprinting\|Phte\|Klf4, Motifs\|Footprinting\|Hsmm\|Klf4, Motifs\|Footprinting\|Hepatocytes\|Klf4, Motifs\|Footprinting\|Helas3\|Klf4, Motifs\|Footprinting\|Osteobl\|Klf4, Motifs\|Footprinting\|Gliobla\|Klf4, Motifs\|Footprinting\|8988t\|Klf4, Motifs\|Footprinting\|Fibrop\|Klf4, Motifs\|Footprinting\|Nhek\|Klf4, Motifs\|Footprinting\|LncapAndro\|CACD, Motifs\|Footprinting\|Hsmmt\|Klf4, Motifs\|Footprinting\|A549\|Klf4, Motifs\|Footprinting\|Hmec\|Klf4, Motifs\|PWM\|\|CACD, Motifs\|Footprinting\|K562\|Klf4, Motifs\|PWM\|\|Klf4, Motifs\|Footprinting\|Melano\|Klf4, Motifs\|Footprinting\|Gm12892\|Klf4, Motifs\|Footprinting\|Myometr\|Klf4, Motifs\|Footprinting\|Gm12891\|Klf4, Motifs\|PWM\|\|Staf, Motifs\|Footprinting\|Helas3Ifna4h\|Klf4, Motifs\|PWM\|\|ZNF143, Motifs\|PWM\|\|SP1, Motifs\|Footprinting\|Panislets\|Klf4, Motifs\|Footprinting\|Gm12878\|Klf4, Motifs\|Footprinting\|Gm19238\|Klf4, Motifs\|Footprinting\|Medullo\|Klf4, Motifs\|Footprinting\|Chorion\|Klf4, Motifs\|Footprinting\|Huh7\|Klf4, Motifs\|PWM\|\|KLF16, Motifs\|Footprinting\|Hepg2\|Klf4, Motifs\|PWM\|\|SP3, Motifs\|Footprinting\|Hpde6e6e7\|Klf4, Motifs\|Footprinting\|Panisd\|Klf4, Motifs\|Footprinting\|H9es\|Klf4, Motifs\|Footprinting\|Gm19239\|Klf4, Motifs\|Footprinting\|Huvec\|Klf4, Motifs\|Footprinting\|Fibrobl\|Klf4, Motifs\|Footprinting\|Mcf7Hypoxlac\|Klf4, Motifs\|Footprinting\|LncapAndro\|Klf4, Motifs\|Footprinting\|Lncap\|Klf4, Motifs\|Footprinting\|H1hesc\|Klf4, Chromatin_Structure\|Hypoxlac\|DNase-seq\|Mcf7, Chromatin_Structure\|\|DNase-seq\|Ipscwru1, Chromatin_Structure\|Estctrl0h\|DNase-seq\|Mcf7, Chromatin_Structure\|\|DNase-seq\|Ips, Chromatin_Structure\|\|DNase-seq\|Mcf7, Chromatin_Structure\|Est100nm1h\|DNase-seq\|Mcf7, Chromatin_Structure\|Hypoxlaccon\|DNase-seq\|Mcf7, Chromatin_Structure\|\|FAIRE\|Nhbe, Chromatin_Structure\|\|DNase-seq\|A549, Chromatin_Structure\|Randshrna\|DNase-seq\|Mcf7, Protein_Binding\|serum_starved_media\|ChIP-seq\|MCF-7\|CTCF, Protein_Binding\|\|ChIP-seq\|HEK293-T-REx\|ZNF263, Protein_Binding\|\|ChIP-seq\|MCF-7\|CTCF, Protein_Binding\|serum_stimulated_media\|ChIP-seq\|MCF-7\|CTCF, Protein_Binding\|vehicle\|ChIP-seq\|MCF-7\|CTCF, Protein_Binding\|estrogen\|ChIP-seq\|MCF-7\|CTCF | 2b |
| rs10208418 | Chromatin_Structure\|Est10nm30m\|DNase-seq\|T47d, Chromatin_Structure\|\|DNase-seq\|H1hesc, Chromatin_Structure\|\|DNase-seq\|Progfib, Chromatin_Structure\|\|DNase-seq\|Huh75, Chromatin_Structure\|\|DNase-seq\|Gm19239, Chromatin_Structure\|\|DNase-seq\|Hah, Chromatin_Structure\|\|DNase-seq\|Ipsnihi7, Chromatin_Structure\|Diffa5d\|DNase-seq\|H7es, Chromatin_Structure\|\|DNase-seq\|Hcm, Chromatin_Structure\|\|DNase-seq\|Hpdlf, Chromatin_Structure\|Diffa9d\|DNase-seq\|H7es, Chromatin_Structure\|\|DNase-seq\|H9es, Chromatin_Structure\|Diff4d\|DNase-seq\|Lhcnm2, Chromatin_Structure\|\|DNase-seq\|Werirb1, Chromatin_Structure\|\|DNase-seq\|Hepg2, Chromatin_Structure\|\|DNase-seq\|Hae, Chromatin_Structure\|Hypoxlac\|DNase-seq\|Mcf7, Chromatin_Structure\|\|DNase-seq\|Ipscwru1, Chromatin_Structure\|\|DNase-seq\|Lhcnm2, Chromatin_Structure\|\|DNase-seq\|Ipsnihi11, Chromatin_Structure\|Estctrl0h\|DNase-seq\|Mcf7, Chromatin_Structure\|\|DNase-seq\|Ips, Chromatin_Structure\|\|DNase-seq\|Medullod341, Chromatin_Structure\|\|DNase-seq\|Medullo, Chromatin_Structure\|\|DNase-seq\|Heartoc, Chromatin_Structure\|\|DNase-seq\|Mcf7, Chromatin_Structure\|\|DNase-seq\|Fibrop, Chromatin_Structure\|Est100nm1h\|DNase-seq\|Mcf7, Chromatin_Structure\|\|DNase-seq\|Hcpe, Chromatin_Structure\|\|DNase-seq\|H7es, Chromatin_Structure\|\|DNase-seq\|Hbmec, Chromatin_Structure\|Hypoxlaccon\|DNase-seq\|Mcf7, Chromatin_Structure\|\|DNase-seq\|Hsmm, Chromatin_Structure\|\|DNase-seq\|T47d, Chromatin_Structure\|\|DNase-seq\|A549, Chromatin_Structure\|Randshrna\|DNase-seq\|Mcf7, Chromatin_Structure\|\|DNase-seq\|Huh7, Chromatin_Structure\|\|DNase-seq\|Helas3, Chromatin_Structure\|\|DNase-seq\|Nt2d1, Chromatin_Structure\|\|DNase-seq\|Hsmmt, Chromatin_Structure\|\|DNase-seq\|Cerebrumfrontaloc, Chromatin_Structure\|Ifna4h\|DNase-seq\|Helas3, Chromatin_Structure\|\|DNase-seq\|Hek293t, Protein_Binding\|\|ChIP-seq\|SK-N-SH\|RAD21, Protein_Binding\|\|ChIP-seq\|HepG2\|RAD21, Protein_Binding\|\|ChIP-seq\|Osteobl\|CTCF, Protein_Binding\|\|ChIP-seq\|ProgFib\|CTCF, Protein_Binding\|serum_starved_media\|ChIP-seq\|MCF-7\|CTCF, Protein_Binding\|\|ChIP-seq\|GM06990\|CTCF, Protein_Binding\|\|ChIP-seq\|HCPEpiC\|CTCF, Protein_Binding\|\|ChIP-seq\|NHEK\|CTCF, Protein_Binding\|\|ChIP-seq\|Gliobla\|CTCF, Protein_Binding\|\|ChIP-seq\|HSMMtube\|CTCF, Protein_Binding\|\|ChIP-seq\|Caco-2\|CTCF, Protein_Binding\|\|ChIP-seq\|HSMM\|CTCF, Protein_Binding\|\|ChIP-seq\|HPAF\|CTCF, Protein_Binding\|\|ChIP-seq\|MCF-7\|CTCF, Protein_Binding\|\|ChIP-seq\|HCM\|CTCF, Protein_Binding\|serum_stimulated_media\|ChIP-seq\|MCF-7\|CTCF, Protein_Binding\|\|ChIP-seq\|GM12872\|CTCF, Protein_Binding\|\|ChIP-seq\|H1-hESC\|CTCF, Protein_Binding\|\|ChIP-seq\|GM12891\|CTCF, Protein_Binding\|\|ChIP-seq\|A549\|CTCF, Protein_Binding\|\|ChIP-seq\|BE2\|CTCF, Protein_Binding\|\|ChIP-seq\|HEK293\|CTCF, Protein_Binding\|\|ChIP-seq\|GM19240\|CTCF, Protein_Binding\|\|ChIP-seq\|GM12878\|CTCF, Protein_Binding\|\|ChIP-seq\|HAc\|CTCF, Protein_Binding\|\|ChIP-seq\|K562\|CTCF, Protein_Binding\|\|ChIP-seq\|HBMEC\|CTCF, Protein_Binding\|\|ChIP-seq\|GM12892\|CTCF, Protein_Binding\|\|ChIP-seq\|GM19238\|CTCF, Protein_Binding\|\|ChIP-seq\|IMR90\|CTCF, Protein_Binding\|\|ChIP-seq\|HepG2\|CTCF, Protein_Binding\|\|ChIP-seq\|HRPEpiC\|CTCF, Protein_Binding\|\|ChIP-seq\|RPTEC\|CTCF, Protein_Binding\|\|ChIP-seq\|H1-hESC\|RAD21, Protein_Binding\|\|ChIP-seq\|WERI-Rb-1\|CTCF, Protein_Binding\|estrogen\|ChIP-seq\|MCF-7\|CTCF | 4 |
| rs72853591 | Chromatin_Structure\|\|DNase-seq\|Cd34mobilized, Protein_Binding\|\|ChIP-seq\|PFSK-1\|TAF1, Protein_Binding\|\|ChIP-seq\|CD34\|SMARCA4 | 4 |
| rs7563889 | Chromatin_Structure\|\|DNase-seq\|Huh75, Chromatin_Structure\|\|DNase-seq\|K562, Chromatin_Structure\|\|DNase-seq\|H9es, Chromatin_Structure\|\|DNase-seq\|Hepg2, Chromatin_Structure\|Estctrl0h\|DNase-seq\|Mcf7, Chromatin_Structure\|\|DNase-seq\|K562G1phase, Chromatin_Structure\|\|DNase-seq\|Mcf7, Chromatin_Structure\|\|DNase-seq\|Cmk, Chromatin_Structure\|Est100nm1h\|DNase-seq\|Mcf7, Chromatin_Structure\|\|DNase-seq\|H7es, Chromatin_Structure\|\|DNase-seq\|A549, Chromatin_Structure\|\|DNase-seq\|Huh7, Chromatin_Structure\|\|DNase-seq\|Be2c, Chromatin_Structure\|\|DNase-seq\|Cd34mobilized, Protein_Binding\|\|ChIP-seq\|AG04450\|CTCF, Protein_Binding\|\|ChIP-seq\|MCF-7\|CTCF, Protein_Binding\|\|ChIP-seq\|HCM\|CTCF, Protein_Binding\|\|ChIP-seq\|H1-hESC\|CTCF, Protein_Binding\|\|ChIP-seq\|BE2\|CTCF, Protein_Binding\|\|ChIP-seq\|HEK293\|CTCF, Protein_Binding\|\|ChIP-seq\|HMF\|CTCF, Protein_Binding\|\|ChIP-seq\|K562\|CTCF, Protein_Binding\|vehicle\|ChIP-seq\|MCF-7\|CTCF, Protein_Binding\|\|ChIP-seq\|GM12873\|CTCF, Protein_Binding\|\|ChIP-seq\|HepG2\|CTCF, Protein_Binding\|estrogen\|ChIP-seq\|MCF-7\|CTCF | 4 |
| rs79103588 | Chromatin_Structure\|\|DNase-seq\|Nhdfneo, Chromatin_Structure\|\|DNase-seq\|Sknmc, Protein_Binding\|\|ChIP-seq\|SK-N-MC\|POLR2A, Protein_Binding\|EWS-FLI1-fusion\|ChIP-seq\|SK-N-MC\|FLI1, Protein_Binding\|EWS-FLI1-fusion\|ChIP-seq\|SK-N-MC\|EWSR1 | 4 |
| rs849573 | Chromatin_Structure\|\|DNase-seq\|Osteobl, Chromatin_Structure\|\|DNase-seq\|Medullo, Protein_Binding\|\|ChIP-seq\|GM12875\|CTCF, Protein_Binding\|\|ChIP-seq\|GM12873\|CTCF, Protein_Binding\|\|ChIP-seq\|HepG2\|CTCF | 4 |
| rs10041963 | Chromatin_Structure\|\|DNase-seq\|Hsmm, Chromatin_Structure\|\|DNase-seq\|Hsmmt, Chromatin_Structure\|\|DNase-seq\|Rpmi7951 | 5 |
| rs10170423 | Chromatin_Structure\|Diffa5d\|DNase-seq\|H7es, Chromatin_Structure\|\|DNase-seq\|Hpdlf, Chromatin_Structure\|\|DNase-seq\|Monocd14ro1746, Chromatin_Structure\|\|DNase-seq\|Cmk, Chromatin_Structure\|\|DNase-seq\|H7es, Chromatin_Structure\|\|DNase-seq\|Monocd14, Chromatin_Structure\|\|DNase-seq\|Nhdfad | 5 |
| rs10208981 | Motifs\|PWM\|\|ZNF410, Chromatin_Structure\|\|DNase-seq\|Th17, Chromatin_Structure\|\|DNase-seq\|Th1 | 5 |
| rs10490124 | Motifs\|PWM\|\|Mafk, Chromatin_Structure\|\|DNase-seq\|Hgf | 5 |
| rs11033879 | Chromatin_Structure\|\|DNase-seq\|Monocd14ro1746, Chromatin_Structure\|\|DNase-seq\|Monocd14 | 5 |
| rs11245920 | Chromatin_Structure\|Est10nm30m\|DNase-seq\|T47d, Chromatin_Structure\|\|DNase-seq\|Adultcd4th1, Chromatin_Structure\|\|DNase-seq\|T47d | 5 |
| rs113659777 | Motifs\|Footprinting\|GM12891\|p53, Chromatin_Structure\|\|DNase-seq\|Gm12891, Chromatin_Structure\|\|DNase-seq\|Progfib, Chromatin_Structure\|\|DNase-seq\|8988t, Chromatin_Structure\|\|DNase-seq\|Hmec, Chromatin_Structure\|\|DNase-seq\|Osteobl, Chromatin_Structure\|\|DNase-seq\|Olfneurosphere, Chromatin_Structure\|Andro\|DNase-seq\|Lncap, Chromatin_Structure\|\|DNase-seq\|Imr90, Chromatin_Structure\|\|DNase-seq\|Gm10248, Chromatin_Structure\|\|DNase-seq\|Huvec, Chromatin_Structure\|\|DNase-seq\|Cd20ro01794, Chromatin_Structure\|\|DNase-seq\|Ips, Chromatin_Structure\|\|DNase-seq\|Heartoc, Chromatin_Structure\|\|DNase-seq\|Medullod341, Chromatin_Structure\|\|DNase-seq\|Medullo, Chromatin_Structure\|\|DNase-seq\|Chorion, Chromatin_Structure\|\|DNase-seq\|Myometr, Chromatin_Structure\|\|DNase-seq\|Gm10266, Chromatin_Structure\|\|DNase-seq\|Gm12892, Chromatin_Structure\|\|DNase-seq\|H7es, Chromatin_Structure\|\|DNase-seq\|Hsmm, Chromatin_Structure\|\|DNase-seq\|Monocd14, Chromatin_Structure\|\|DNase-seq\|Lncap, Chromatin_Structure\|\|DNase-seq\|Panislets, Chromatin_Structure\|\|DNase-seq\|Gm13976 | 5 |
| rs113936625 | Motifs\|PWM\|\|MAFK, Motifs\|PWM\|\|Mafk, Motifs\|PWM\|\|Mafb, Motifs\|PWM\|\|MAFB, Motifs\|PWM\|\|NRL, Chromatin_Structure\|\|DNase-seq\|Hbvp, Chromatin_Structure\|\|DNase-seq\|Fibrobl, Chromatin_Structure\|Lentimyod\|DNase-seq\|Fibroblgm03348, Chromatin_Structure\|\|DNase-seq\|Fibroblgm03348, Chromatin_Structure\|\|FAIRE\|Nhbe | 5 |
| rs115071514 | Chromatin_Structure\|\|DNase-seq\|Monocd14ro1746, Chromatin_Structure\|\|DNase-seq\|Monocd14 | 5 |
| rs118052517 | Protein_Binding\|\|ChIP-seq\|K562\|CEBPB | 5 |
| rs184239416 | Motifs\|Footprinting\|H1_ES\|, Motifs\|Footprinting\|GM12891\|p53, Chromatin_Structure\|\|DNase-seq\|Gm12891, Chromatin_Structure\|\|DNase-seq\|Progfib, Chromatin_Structure\|\|DNase-seq\|8988t, Chromatin_Structure\|\|DNase-seq\|Hmec, Chromatin_Structure\|\|DNase-seq\|Osteobl, Chromatin_Structure\|\|DNase-seq\|Olfneurosphere, Chromatin_Structure\|Andro\|DNase-seq\|Lncap, Chromatin_Structure\|\|DNase-seq\|Imr90, Chromatin_Structure\|\|DNase-seq\|Gm10248, Chromatin_Structure\|\|DNase-seq\|Huvec, Chromatin_Structure\|\|DNase-seq\|Cd20ro01794, Chromatin_Structure\|\|DNase-seq\|Ips, Chromatin_Structure\|\|DNase-seq\|Heartoc, Chromatin_Structure\|\|DNase-seq\|Medullod341, Chromatin_Structure\|\|DNase-seq\|Medullo, Chromatin_Structure\|\|DNase-seq\|Chorion, Chromatin_Structure\|\|DNase-seq\|Myometr, Chromatin_Structure\|\|DNase-seq\|Gm10266, Chromatin_Structure\|\|DNase-seq\|Gm12892, Chromatin_Structure\|\|DNase-seq\|H7es, Chromatin_Structure\|\|DNase-seq\|Hsmm, Chromatin_Structure\|\|DNase-seq\|Monocd14, Chromatin_Structure\|\|DNase-seq\|Lncap, Chromatin_Structure\|\|DNase-seq\|Panislets, Chromatin_Structure\|\|DNase-seq\|Gm13976 | 5 |
| rs41392245 | Motifs\|PWM\|\|TCFAP2A, Motifs\|PWM\|\|TFAP2B, Motifs\|PWM\|\|Tcfap2a, Motifs\|PWM\|\|TFAP2C, Chromatin_Structure\|\|DNase-seq\|Gm12891, Chromatin_Structure\|\|DNase-seq\|Progfib, Chromatin_Structure\|\|DNase-seq\|8988t, Chromatin_Structure\|\|DNase-seq\|H1hesc, Chromatin_Structure\|\|DNase-seq\|Hmec, Chromatin_Structure\|\|DNase-seq\|Osteobl, Chromatin_Structure\|\|DNase-seq\|K562, Chromatin_Structure\|\|DNase-seq\|Olfneurosphere, Chromatin_Structure\|\|DNase-seq\|Gm13977, Chromatin_Structure\|Andro\|DNase-seq\|Lncap, Chromatin_Structure\|\|DNase-seq\|Imr90, Chromatin_Structure\|\|DNase-seq\|Hepg2, Chromatin_Structure\|\|DNase-seq\|Hsmmfshd, Chromatin_Structure\|\|DNase-seq\|Cd20ro01794, Chromatin_Structure\|\|DNase-seq\|Ips, Chromatin_Structure\|\|DNase-seq\|Medullod341, Chromatin_Structure\|\|DNase-seq\|Medullo, Chromatin_Structure\|\|DNase-seq\|Chorion, Chromatin_Structure\|\|DNase-seq\|Hepatocytes, Chromatin_Structure\|\|DNase-seq\|Gm10266, Chromatin_Structure\|\|DNase-seq\|Gm12892, Chromatin_Structure\|\|DNase-seq\|Frontalcortexoc, Chromatin_Structure\|\|DNase-seq\|H7es, Chromatin_Structure\|\|DNase-seq\|Hsmm, Chromatin_Structure\|\|DNase-seq\|Psoasmuscleoc, Chromatin_Structure\|\|DNase-seq\|Huh7, Chromatin_Structure\|\|DNase-seq\|Cd20ro01778, Chromatin_Structure\|\|DNase-seq\|Monocd14, Chromatin_Structure\|\|DNase-seq\|Lncap, Chromatin_Structure\|\|DNase-seq\|Hsmmt | 5 |
| rs55989629 | Chromatin_Structure\|\|DNase-seq\|Helas3 | 5 |
| rs57185274 | Motifs\|PWM\|\|Bhlhb2, Motifs\|PWM\|\|Zscan4, Chromatin_Structure\|\|DNase-seq\|Th1 | 5 |
| rs66689811 | Chromatin_Structure\|Ifna4h\|FAIRE\|Helas3, Chromatin_Structure\|\|DNase-seq\|Helas3, Chromatin_Structure\|Ifna4h\|DNase-seq\|Helas3 | 5 |
| rs72846811 | Chromatin_Structure\|\|DNase-seq\|Chorion, Chromatin_Structure\|\|DNase-seq\|Helas3, Chromatin_Structure\|Ifna4h\|DNase-seq\|Helas3 | 5 |
| rs75201173 | Motifs\|PWM\|\|NF-AT, Protein_Binding\|\|ChIP-seq\|U2OS\|SETDB1 | 5 |
| rs75358328 | Protein_Binding\|\|ChIP-seq\|MCF-7\|E2F1 | 5 |
| rs7570091 | Chromatin_Structure\|ra\|DNase-seq\|Sknsh, Chromatin_Structure\|\|DNase-seq\|Sknsh | 5 |
| rs79503131 | Motifs\|PWM\|\|VDR, Motifs\|PWM\|\|Rfx4, Motifs\|PWM\|\|Rfxdc2, Chromatin_Structure\|\|DNase-seq\|Wi38, Chromatin_Structure\|\|DNase-seq\|Hipe, Chromatin_Structure\|\|DNase-seq\|Hrpe, Chromatin_Structure\|\|DNase-seq\|Hae, Chromatin_Structure\|\|DNase-seq\|Hnpce, Chromatin_Structure\|\|DNase-seq\|Myometr, Chromatin_Structure\|\|DNase-seq\|Hcpe, Chromatin_Structure\|\|DNase-seq\|Hbmec, Chromatin_Structure\|\|DNase-seq\|Hasp, Chromatin_Structure\|\|DNase-seq\|Skmc | 5 |
| rs849567 | Chromatin_Structure\|Znfa41c6\|DNase-seq\|K562, Chromatin_Structure\|\|DNase-seq\|Gm12891, Chromatin_Structure\|\|DNase-seq\|Hconf, Chromatin_Structure\|\|DNase-seq\|Melano, Chromatin_Structure\|\|DNase-seq\|Hipe, Chromatin_Structure\|\|DNase-seq\|K562, Chromatin_Structure\|\|DNase-seq\|Stellate, Chromatin_Structure\|\|DNase-seq\|Hepg2, Chromatin_Structure\|\|DNase-seq\|Gm12892 | 5 |
| rs849577 | Motifs\|PWM\|\|Zfp105, Motifs\|PWM\|\|Elf3, Motifs\|PWM\|\|Srf, Motifs\|PWM\|\|Tcfap2e, Chromatin_Structure\|ra\|DNase-seq\|Sknsh, Chromatin_Structure\|\|DNase-seq\|Medullo, Chromatin_Structure\|\|DNase-seq\|Sknsh | 5 |
| rs849578 | Motifs\|PWM\|\|HIC1, Chromatin_Structure\|\|DNase-seq\|Huvec | 5 |
| rs10919229 | Motifs\|PWM\|\|Tcf3, Motifs\|PWM\|\|Zfp105, Motifs\|PWM\|\|Elf3, Motifs\|PWM\|\|Srf, Motifs\|PWM\|\|BCL6, Motifs\|PWM\|\|Tcfap2e, Chromatin_Structure\|\|FAIRE\|K562 | 6 |
| rs114372180 | Motifs\|PWM\|\|Arid5a | 6 |
| rs137985802 | Motifs\|PWM\|\|COMP1 | 6 |
| rs144499291 | Motifs\|PWM\|\|Zfp281, Motifs\|PWM\|\|KROX | 6 |
| rs189340111 | Motifs\|PWM\|\|MSX1, Motifs\|PWM\|\|POU6F1, Motifs\|PWM\|\|BARX1, Motifs\|PWM\|\|Zfp128, Motifs\|PWM\|\|Tbp, Motifs\|PWM\|\|Oct-1 | 6 |
| rs191508159 | Motifs\|PWM\|\|Freac-3 | 6 |
| rs1992368 | Motifs\|PWM\|\|IPF1 | 6 |
| rs6861945 | Motifs\|PWM\|\|FOXP3 | 6 |
| rs77957383 | Motifs\|PWM\|\|HMGIY, Motifs\|PWM\|\|Sox4, Motifs\|PWM\|\|Sox11 | 6 |
| rs80143982 | Motifs\|PWM\|\|Barhl1, Motifs\|PWM\|\|Msx-3 | 6 |
| rs849583 | Motifs\|PWM\|\|Gm397, Motifs\|PWM\|\|FOXP1, Motifs\|PWM\|\|RREB1 | 6 |
| rs10072716 | No data | 7 |
| rs111452409 | No data | 7 |
| rs111875224 | No data | 7 |
| rs114273249 | No data | 7 |
| rs114288750 | No data | 7 |
| rs11684178 | No data | 7 |
| rs11956277 | No data | 7 |
| rs12468670 | No data | 7 |
| rs13385591 | No data | 7 |
| rs13429699 | No data | 7 |
| rs13430743 | No data | 7 |
| rs13432631 | No data | 7 |
| rs142402253 | No data | 7 |
| rs148619316 | No data | 7 |
| rs150958961 | No data | 7 |
| rs17801458 | No data | 7 |
| rs183898825 | No data | 7 |
| rs1998725 | No data | 7 |
| rs61564830 | No data | 7 |
| rs6718590 | No data | 7 |
| rs6863067 | No data | 7 |
| rs71560740 | No data | 7 |
| rs73963540 | No data | 7 |
| rs74871811 | No data | 7 |
| rs75148923 | No data | 7 |
| rs75349221 | No data | 7 |
| rs75372391 | No data | 7 |
| rs7562208 | No data | 7 |
| rs7563070 | No data | 7 |
| rs7600883 | No data | 7 |
| rs7602404 | No data | 7 |
| rs76908315 | No data | 7 |
| rs79931187 | No data | 7 |
| rs830612 | No data | 7 |
| rs849572 | No data | 7 |

^a^ Lower score indicates more evidence that the eQTL was detected as a functional region. For example, the score of 2b is likely to affect binding of functional region (transcription factor binding, any motif, DNase footprint, and DNase peak). The scores of 4, 5, and 6 have minimal binding evidence of functional region. The score of 7 indicates no functional evidence.
